# When the world collapses: changed worldview and social reconstruction in a traumatized community

**DOI:** 10.3402/ejpt.v5.24098

**Published:** 2014-09-11

**Authors:** Dinka Corkalo Biruski, Dean Ajdukovic, Ajana Löw Stanic

**Affiliations:** Department of Psychology, Faculty of Humanities and Social Sciences, University of Zagreb, Croatia

**Keywords:** War-related trauma, worldview changes, community social reconstruction, intergroup relations

## Abstract

**Background:**

Traumatic experience can affect the individual's basic beliefs about the world as a predictable and safe place. One of the cornerstones in recovery from trauma is reestablishment of safety, connectedness, and the shattered schema of a worldview.

**Objective:**

This study explored the role of negatively changed worldview in the relationship between war-related traumatization and readiness for social reconstruction of intergroup relations in a post-conflict community measured by three processes: intergroup rapprochement, rebuilding trust, and need for apology. It was hypothesized that more traumatized people are less supportive of social reconstruction and that this relationship is mediated by the changed worldview.

**Method:**

The study included a community random sample of 333 adults in the city of Vukovar, Croatia, that was most devastated during the 1991–1995 war. Six instruments were administered: Stressful Events Scale, Impact of Event Scale-Revised, Changed Worldview Scale, and three scales measuring the post-conflict social reconstruction processes: Intergroup Rapprochement, Intergroup Trust and Need for Apology.

**Results:**

Mediation analyses showed that the worldview change fully mediated between traumatization and all three aspects of social reconstruction.

**Conclusions:**

In a population exposed to war traumatization the worldview change mediates post-conflict social recovery of community relations.

People generally believe that the world is a reasonably safe, predictable, benevolent, and meaningful place. This set of beliefs is at the core of our fundamental assumptions (‘assumptive world’) about who we are, what is the world around us and how we make sense about those two (Janoff-Bulman, 1992). In a daily routine, when things happen as expected, our assumptive world helps us to perceive a balanced contingency between what we do and what we get as an outcome. However, when this routine is severely disturbed, as in the case of trauma, this may have a devastating impact on this balance and cause our fundamental assumptions to be ‘shattered’ (Janoff-Bulman, 1989). Trauma survivors with profound changes of their belief system experience also more severe mental health symptoms, like complicated bereavement (Currier, Holland, & Neimeyer, 2009), posttraumatic stress disorder (PTSD) (Goldberg & Matheson, 2005), and depression (Janoff-Bulman, 1989; Lilly, Valdez, & Graham-Bermann, 2011). Although some findings question the link between worldview damage and trauma symptoms (Edmondson et al., 2011), the major coping task for trauma survivors, as Janoff-Bulman (1992) pointed out, is to restore the core belief system and become functional again.

An injury makes people vulnerable but also motivated to withdraw from the source of the hurt. As we have noticed elsewhere (Corkalo Biruski & Penic, 2014), this is more easily accomplished in the case of individual trauma, when the source of hurt is clearly identifiable. In the context of intergroup conflict, sources of the hurt may be many, and responsibility for one's suffering could be assigned to the whole group instead of an individual. Although negative relations between traumatic experiences and relations towards the out-group that is held responsible for the injury can be assumed, this is more intuitive than an established fact (cf. Canetti-Nisim, Halperin, Sharvit, & Hobfoll, 2009). When such a link was found, the correlation was small (Corkalo Biruski & Penic, 2014; Hewstone et al., 2004; Myers, Hewstone, & Cairns, 2009; Staub, Pearlman, Gubin, & Hagengimana, 2005).

Since our assumptive system includes core beliefs about self and other human beings, human-induced victimization is particularly harmful for maintaining the healthy worldview (Lilly, Valdez, & Graham-Bermann, 2011; Wickie & Marwit, 2000), especially in the context of massive violence and gross violation of human rights (Magwaza, 1999; Sandole & Auerbach, 2013). Because the assumptive world system serves as an inner protective mechanism that allows us to perceive the outside world as safe and secure, it may have a crucial role in linking human-induced trauma of war and relations to the former belligerent out-group. This relationship has not yet been studied in the context of intergroup relations, though some studies showed that feeling insecure under circumstances of an ongoing out-group threat may have a negative impact on a variety of attitudes and behaviors towards the conflicting groups (e.g., Canetti, Hall, Rapaport, & Wayne, 2013). In the present study, the threat caused by war experiences implies a deep intra-psychological change in affected individuals whose *general* worldview has been threatened (and not only the views about the out-group seen as the major perpetrator). Hence, the present research contributes to the literature by making a link between trauma-related intra-psychological changes of core beliefs and consequential social tendencies to improve relations with former enemies. Specifically, the study tests the hypothesis that the changed worldview mediates the relationship between war-related traumatization and willingness to engage in more positive social relations with the conflicting out-group. We refer to this engagement as the social reconstruction process (Ajdukovic, 2004) and follow Nadler's (2002) notion that coming closer between conflicting groups could be instrumental and socio-emotional. While instrumental reconciliation implies cooperative relationships that gradually lead to intergroup trust, socio-emotional processes deal with conflict-related emotions through truth telling, apology, and forgiveness (Nadler, 2002). Hence, we propose that post-conflict social reconstruction includes intergroup rapprochement, rebuilding of intergroup trust, and need for apology from the out-group. We expect that changes in the assumptive world will mediate the relationship between trauma and all three aspects of social reconstruction.

## Method

### Participants

Data were collected in 2008 on a community random sample, obtained by a random-walk sampling technique. Participants included 333 women (64%) and men, 18–65 years old, living in the city of Vukovar, Croatia, that experienced massive violence and human and material losses in 1991–1995 war. About 63% of the sample were ethnic Croats, and the rest were Serbs. As part of a larger study, participants were assessed in an individual session at their homes. A trained senior psychology student assisted written completion of the questionnaire. A signed statement of informed consent was obtained from every participant. The Ethical Review Board of the Department of Psychology University of Zagreb approved the study.

### Measures

Six instruments relevant for the present study were administered in local languages. The Stressful Events Scale (SES; Ajdukovic, Ajdukovic, & Kulenovic, 1993) is a 26-item measure of exposure to war-related events. Participants indicated whether they experienced the event or not. In the present, analysis responses on 12 items (*α*=0.71) that describe life-threatening and traumatizing events were summed up, meeting the requirements for Criterion A for a PTSD diagnosis (e.g., exposure to bombardment, shooting, imprisonment).

The Impact of Event Scale-Revised (IES-R; Weiss & Marman, 1997) is a widely used 22-item scale to assess reactions to a traumatic event. Participants reported their reactions during the past week with respect to what they experienced during the war (e.g., intrusive thoughts, nightmares, avoidance of feelings, anger, irritability …). The clinical cutoff score on the IES-R is 22 (Rash, Coffey, Baschnagel, Drobes, & Saladin, 2008). The total score was used as a measure of traumatization (*α*=0.96). The translated scale has been shown to retain the original metric properties (Morina et al., 2010) and has been widely used on different war-affected samples in the former Yugoslavia (e.g., Priebe et al., 2013).

The Changed Worldview Scale (CWS; Corkalo & Ajdukovic, 2002) measures changes attributed to war-related experiences in a set of basic beliefs about the world as a just, safe and trustful place. It is a unidimensional 10-item scale, with good internal consistency (*α*=0.86) and factor loadings above 0.30. Item examples are: *War has made me stop believing in what I believed before*, or *Since the war I feel the world has not been a safe place anymore*. Participants endorsed each statement on a five-point scale, with higher score indicating more profound worldview changes.

A tendency to re-establish relations with the former belligerent group was measured by three scales (Ajdukovic, Corkalo Biruski, & Löw, in preparation). The Intergroup Rapprochement Scale consists of nine statements (*α*=0.87) that indicate willingness for more positive intergroup relations (e.g., *I think it is important for our children to cooperate*; or *I believe that the other side also suffered during the war*.). The Intergroup Trust Scale also comprises nine-items (*α*=0.83) describing beliefs about rebuilding trust between groups (e.g., *I think that trust between Croats and Serbs has been lost forever*; or *I don't think it is possible to ever overcome the wounds from the past war*). The Need for Apology Scale includes three items (*α*=0.60) that describe beliefs that apology is necessary for improving intergroup relations (e.g., *It is important for me that the other side offers an apology;* or *I would like the other side to show remorse for our victims*). Response format was from 1 (strongly disagree) to 5 (strongly agree), and mean scores were calculated for each scale. The scales were validated on a large sample of 924 participants from five different war-affected communities (Ajdukovic et al., in preparation).

### Data analyses

To validate the mediation effects, we performed structural equation modeling analyses using MPlus 6.0 SEM software (Muthén & Muthén, 2010). To address multivariate nonnormality, we used a robust form of maximum likelihood estimation method (MLR). In keeping with common practice, model fit was evaluated by Chi-squared statistic, root mean squared error of approximation, comparative fit index, Tucker-Lewis index, and standardized root mean square residual; using the recent cutoff values guidelines (Hu & Bentler, 1999). Traumatization, worldview change and three aspects of social reconstruction were modeled as latent variables, with scale items as indicators. Analyses were done in three steps, testing: 1) measurement models, 2) structural model with direct effects, 3) structural model with both direct and indirect effects, where worldview change was examined as a possible mediator.

## Results

An average exposure to war-related experiences was 3.86 events (SD=2.33), ranging from 1 to 10 events. Nineteen participants who reported no traumatic exposure were excluded from further analyses. The final sample consisted of 314 participants. About 62% of the sample experienced one to four war events. Only two participants experienced 10 events and, although being outliers, we included them for the obvious relevance of their experience. The participants reported high levels of trauma symptoms attributed to wartime experiences almost 15 years after exposure: 56.4% of respondents scored higher than the clinical cutoff score on the IES-R. The correlation between war-related exposure and IES-R scores was 0.36, *p*<0.01. After establishing high war-related trauma exposure of our sample, the IES-R scores were further used as a measure of traumatization. Descriptive statistics and Pearson's correlations among variables are presented in Table 1. Higher level of traumatization was associated with increased change of worldview (0.45). Worldview change was negatively correlated with intergroup rapprochement (−0.21) and intergroup trust (−0.52), and positively with a need for apology (0.18). While trust was associated both with rapprochement (0.58) and need for apology (−0.30), rapprochement and need for apology were not significantly correlated.

**Table 1:** Descriptive statistics and Pearson's correlations among variables modeled in structural equation modeling (*N=*314)

Variable	*M*	SD	1	2	3	4	5
1. Traumatization	30.95	24.99	–				
2. Worldview change	3.28	0.94	0.45**	–			
3. Intergroup rapprochement	4.29	0.77	−0.18**	−0.21**	–		
4. Intergroup trust	3.35	0.98	−0.35**	−0.52**	0.58**	–	
5. Need for apology	2.02	1.00	0.21**	0.18**	−0.09	−0.30**	–

Note: *M=*mean; SD=standard deviation

** *p*<0.01.

**Fig. 1:**
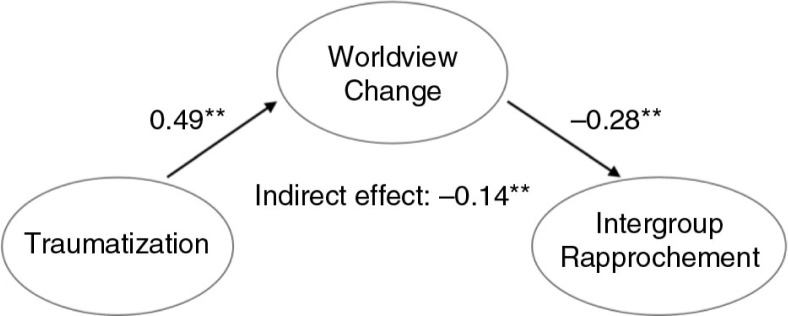
Final structural model of the mediating effect of worldview change on the association between war-related traumatization and intergroup rapprochement (*N=*314). Overall model fit: *χ*^2^(df)=1265.074 (766); *p*<0.01; *χ*^2^/df=1.65; CFI=0.93; TLI=0.92; RMSEA=0.046; *SRMR*=0.062. Note: Only latent variables and standardized parameter estimates are shown; **p<0.01.

**Fig. 2:**
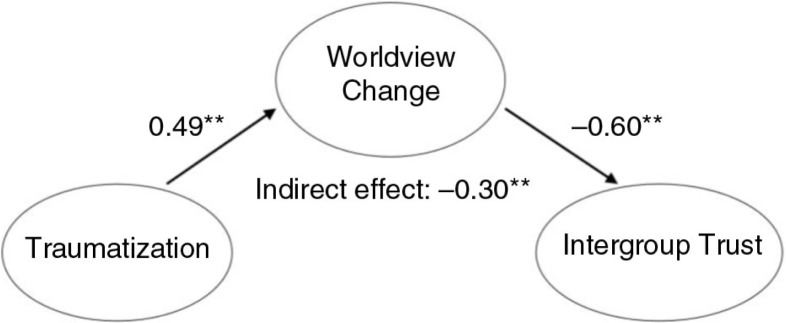
Final structural model of the mediating effect of worldview change on the association between war-related traumatization and intergroup trust (*N=*314). Overall model fit: *χ*^2^(df)=1272.459 (766); *p*<0.01; *χ*^2^/df=1.66; CFI=0.93; TLI=0.92; RMSEA=0.046; SRMR=0.062. Note: Only latent variables and standardized parameter estimates are shown; **p<0.01.

**Fig. 3:**
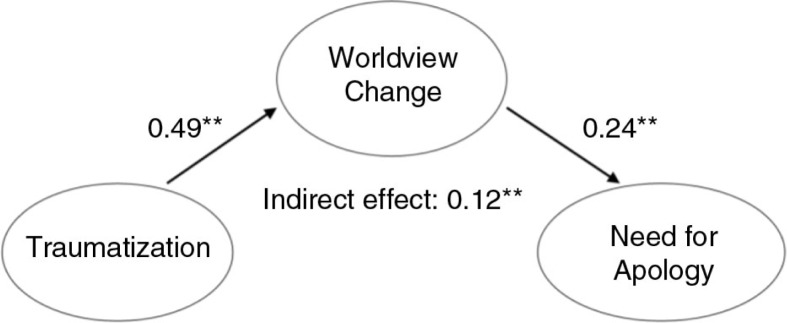
Final structural model of the mediating effect of worldview change on the association between war-related traumatization and need for apology (*N* = 314). Overall model fit: *χ*^2^(df)=919.262 (547); *p*<0.01; *χ*^2^/df=1.68; CFI=0.94; TLI=0.93; RMSEA=0.047; SRMR=0.060. Note: Only latent variables and standardized parameter estimates are shown; **p<0.01.

Results of structural equation modeling are summarized in Figs. 1–3. Final structural models, with war-related traumatization as a predictor variable, worldview change as a mediator and three aspects of social reconstruction as dependent variables, demonstrated good fit.

As shown in Fig. 1, traumatization had a significant indirect effect on intergroup rapprochement through worldview change (*p*<0.01). A value of −0.14 indicated a medium size indirect effect (Preacher & Kelley, 2011).

As shown in Fig. 2, traumatization had a large size indirect effect (−0.30; *p*<0.01) on intergroup trust through worldview change. Similarly, traumatization had a medium size indirect effect (0.12; *p*<0.01) on need for apology through worldview change (Fig. 3).

Direct paths from traumatization to each of the dependent latent variables were non-significant (*p*>0.05), indicating full mediation effects.

## Discussion

The present study tested the hypothesis that worldview change mediates relationship between war-related trauma symptoms and relations with the former belligerent group. Results supported the role of the worldview change as the mediator when three components of social reconstruction were indicators of intergroup relations. More severe war-related trauma symptoms were linked to a more profound worldview change, which was in turn associated with people being less ready to become closer with the members of the group with which they had been in violent conflict, less engaging in trustful relations and more demanding of an apology from the ‘other side.’ The mediating effect of the worldview change was the largest between war-related trauma symptoms and intergroup trust. This may indicate that (intergroup) trust is most seriously violated by trauma inflicted during intergroup violence, as suggested by intergroup reconciliation literature (Ajdukovic, 2004; Bar-Tal, 2013; Nadler, 2002). The role of cognitive schema about others has been recognized as largely responsible for difficulties in (re)establishing intergroup trust after the conflict (Hewstone et al., 2008), indicating that establishing the trust may be only a precondition and not a final outcome in intergroup reconciliatory processes after trauma (cf. Schwegler & Smith, 2012). With restoration of trust more complex processes of social healing may be possible (Nadler & Liviatan, 2004). It is for further research to disentangle interdependencies and possible causal links among these processes.

Our findings also indicate, though with less strength, that the changed worldview may be crucial for other aspects of social reconstruction as well—for intergroup rapprochement and need for apology as necessities for improving the post-conflict relations between groups.

The study corroborates that experience of trauma measured by the symptoms it caused change the general beliefs in people's benevolence and in the world as a reasonably safe place. By doing so trauma does not impair only human psychological recovery but it also ‘shatters’ capacity to recover socially—to trust others and rebuild relations with people who were on the other side of the conflict. In sum, it seems that trauma does not affect one's readiness for social reconstruction directly; its influence is more indirect—by changing the belief system—which further influences social behaviors. It may imply that unless core beliefs are not restored, the social recovery in a community devastated by conflict may not be likely, which has important practical and policy oriented implications for mitigating the effects of war distress and trauma (Hobfoll et al., 2007).

To the best of our knowledge, this is the first study that examines the role of the changed personal worldview in recovery of social relations between groups after massive trauma. Another strength is that both instrumental (intergroup rapprochement) and socio-emotional relations (trust and need for apology) with the out-group have been considered (Nadler, 2002).

The present study has also some limitations. First, the data were retrospective and with no control group, so we cannot be certain that the measured worldview change was caused by trauma. Nevertheless, the same limitation encumbers most studies in the field (cf. Park, 2010). Second, though the major outcome measure, i.e., social reconstruction has been validated in different war-affected communities in context of the same conflict, it may be useful to corroborate its validity in other conflict settings as well. Third, the assessment was done well after the trauma exposure had happened. However, high level of ongoing trauma distress years after exposure indicates that the impact of war remained present in the life of the study participants (cf. Magwaza, 1999). A long period of living in post-war detrimental life conditions might have also influenced the participants’ worldview changes besides the war-related trauma itself. The cross-sectional design does not allow us to answer these questions. It is for further research to address these issues along with exploration of other social consequences of worldview changes after exposure to war-related trauma.
